# Potential of Incorporation of Antimicrobial Plant Phenolics Into Polyolefin-Based Food Contact Materials to Produce Active Packaging by Melt-Blending: Proof of Concept With Isobutyl-4-Hydroxybenzoate

**DOI:** 10.3389/fchem.2019.00148

**Published:** 2019-03-22

**Authors:** Amandine Cottaz, Lynda Bouarab, Justine De Clercq, Nadia Oulahal, Pascal Degraeve, Catherine Joly

**Affiliations:** Université de Lyon, Université Claude Bernard Lyon 1, ISARA Lyon, BioDyMIA (Bioingénierie et Dynamique Microbienne aux Interfaces Alimentaires), Equipe Mixte d'Accueil n°3733, IUT Lyon 1, Technopole Alimentec, Bourg-en-Bresse, France

**Keywords:** antimicrobial, active, food, packaging, polyolefins, release, migration, activity

## Abstract

There is an increasing interest for active food packaging incorporated with natural antimicrobial agents rather than synthetic preservatives. However, most of plastics for direct contact with food are made of polyolefins, usually processed by extrusion, injection, or blow-molding methods while most of natural antimicrobial molecules are thermolabile compounds (e.g., essential oils). Therefore, addition of plant phenolics (with low volatility) to different polyolefins might be promising to design active controlled release packaging processed by usual plastic compounding and used for direct contact with food products. Therefore, up to 2% (wt/wt) of isobutyl-4-hydroxybenzoate (IBHB) was mixed with 3 polyolefins: EVA poly(ethylene-co-vinyl acetate), LLDPE (Linear Low Density Polyethylene), and PP (PolyPropylene) by melt-blending from 75 to 170°C and then pelletized in order to prepare heat-pressed films. IBHB was chosen as an antibacterial phenolic active model molecule against *Staphylococcus aureus* to challenge the entire processing. Antibacterial activity of films against *S. aureus* (procedure adapted from ISO 22196 standard) were 4, 6, and 1 decimal reductions in 24 h for EVA, LLDPE, and PP films, respectively, demonstrating the preservation of the antibacterial activity after melt processing. For food contact materials, the efficacy of antimicrobial packaging depends on the release of the antimicrobial molecules. Therefore, the three types of films were placed at 23°C in 95% (v/v) ethanol and the release rates of IBHB were monitored: 101 ± 1%, 32 ± 7%, and 72 ± 9% at apparent equilibrium for EVA, LLDPE, and PP films, respectively. The apparent diffusion coefficients of IBHB in EVA and PP films were 2.8 ± 0.3 × 10^−12^ and 4.0 ± 1.0 × 10^−16^ m^2^s^−1^. For LLDPE films, IBHB crystals were observed on the surface of films by SEM (Scanning Electron Microscopy): this blooming effect was due the partial incompatibility of IBHB in LLDPE and its fast diffusion out of the polymer matrix onto the film surface. In conclusion, none of these three materials was suitable for a relevant controlled release packaging targeting the preservation of fresh food, but a combination of two of them is promising by the design of a multilayer packaging: the release could result from permeation through an inner PE layer combined with an EVA one acting as a reservoir.

## Introduction

Active food packaging incorporated with antimicrobial molecules are actually deeply studied face to the following dilemma: the consumer demand for natural, minimally processed, or ready-to-eat fresh food (e.g., free from synthetic preservatives) and the problem of food waste induced by microbial spoilage. In that context, active materials can be designed to control over the time (i.e., food shelf life) the release of “natural” antimicrobial substances useful to maintain both quality and safety of food (Bhanu et al., 2015). This issue raises the tricky question of the design of relevant packaging systems, able to be further taken up industrially. Examination of literature reveals that most of active packaging films were prepared by the solvent casting technique, when made from biodegradable polymers or polysaccharides while most of industrial packaging films (polyolefins, polyesters, polystyrene, polyamides) are prepared by extrusion, except coatings for dedicated studies (Del Nobile et al., 2009; Akrami et al., 2015; Gherardi et al., 2016; Wrona et al., 2017; Wu et al., 2018). This issue includes: (i) the selection of packaging polymers which must fulfill the technical specifications met in the packaging industry like easy processing at the molten state with existing facilities (melt-blending), transparency, machinability, sealing ability, and food contact grade (Han and Floros, 1997; Siracusa, 2016). Indeed, potential matrices actually used by industries are most often apolar or semi-polar polymers like polyolefins often used as food contact layers notably for the sealing and copolyesters; (ii) the selection of the antimicrobial additives which have to be natural or agro-sourced candidates, available and of low price, compatible with the polymeric matrices selected above. In fact, the issue rests on the “natural” antimicrobial substance properties which are generally polar, and sometimes even water-soluble, that is to say probably “partially compatible” with the matrices. As stated, the compatibility includes the question of the level of solubility of such substances within the polymer (Nouman et al., 2017) as well as a relevant thermal stability, and low thermal volatility during the process of packaging elaboration (Brody et al., 2001; Colak et al., 2015; Lucera et al., 2016) which can be improved using encapsulation methods (Wrona et al., 2017); (iii) next is the amount of antimicrobial molecules to be incorporated and this point is linked to their solubility, or limit of solubility, but this quantity is often unknown. In literature, the incorporated percentages are generally in between 0.5 and up to 15% wt/wt (Del Nobile et al., 2009; Ramos et al., 2012; Torres et al., 2014), or even more (up to 30%) when embedded in polysaccharides, proteins, or biodegradable polymers (Del Nobile et al., 2008) in order to reach either the MIC (Minimum Inhibitory Concentration), the MBC (Minimum Bactericidal Concentration) [or acceptable daily intake (Quintavalla and Vicini, 2002; Hauser et al., 2016; Suppakul, 2016)] or basically for detection purposes (to increase the experimental sensitivity); (iv) once the binary systems selected, the release can be assessed and/or controlled as a function of time playing with food simulants as receptor media (considering and/or adapted from EC/10/2011), system geometry and external conditions. In fact, both kinetics and amounts of released antimicrobial substances are studied considering their possible elimination during the process, as stated before. In this field, methodologies and know-how can be learnt from literature about food safety regarding polymer additives able to be transferred to food (Figge, 1980; EC, 2011); (v) the final point deals with the control of the effective antimicrobial activity of the substances after material processing and the feasibility of contact with real food to evaluate any enhanced quality and/or shelf life (Colak et al., 2015; Kanatt and Chawla, 2017; Kaewprachu et al., 2018).

As already stated, amongst packaging polymers, polyolefins like polypropylene (PP), polyethylene (PE) and copolymers (EVA or ethylene vinyl acetate copolymer) are good candidates for hosting the antimicrobial substances. They can be used as monolayer packaging for PP and PE (for food with very short shelf life as they are not gas barrier polymers to retain modified atmosphere or limit oxygen ingress) or more interestingly as food contact layer in multilayer packaging for PP, PE, and EVA. EVA can be moreover used as an inner layer (adhesive). Indeed, their transformation temperatures (above melting temperatures) can be tailored to be adapted to antimicrobial substances stability (Pinheiro et al., 2018) choosing from PP (highest melting temperature) to EVA (lowest melting temperature). Moreover, for EVA it is possible to tune the copolymerization rate (vinyl acetate content) to modulate its melting temperature, polarity, and crystallinity rate (Brogly et al., 1997) to play with different levels of additive incorporation.

As antimicrobial substances, plant phenolics are good candidates to be added (Sethi and Gupta, 2016) with low volatility, a rather good thermal stability and structures sometimes close to those of antioxidants added in the formulation of polyolefins (Dopico-García et al., 2007). Phenolics constitute an available source of phenols with diverse structures and strong antimicrobial properties (Janssen et al., 1987; Wen et al., 2003; Pinheiro et al., 2018).

This article presents the results of a feasibility study considering the potential of incorporation of antimicrobial plant phenolics into polyolefin-based food contact materials to produce active packaging by melt-blending.

A proof of concept is proposed with isobutyl-4-hydroxybenzoate (or IBHB) as model phenolic. Indeed, IBHB is a precursor used to synthesize lipophilic alkyl parabens as additives for foods and flavor ingredients. It was also used as a preservative in cosmetic products before its use was banned in Europe in 2014 (Commission Regulation (EU) no 358/2014^1^), adhesives (Vosmann et al., 2008). IBHB (an ester of benzoic acid) was chosen after a preliminary screening of different families of plant phenolics (Bouarab, 2018) which were bactericidal against a *S. aureus* strain. *S.aureus* is a Gram-positive coccus, known as being one the most common causes of food-borne diseases, generally caused by enterotoxins. Its presence in food is attributed to insufficient hygiene when handling the food product (Da Silva Malheiros et al., 2010). IBHB was chosen for its relative hydrophobicity (as estimated by its octanol-water partition coefficient (log Po/w = 2.31) and its molecular weight (MW = 194 g.mol^−1^) which were close to the log P o/w and MW values of many plant phenolics which were also active against *S. aureus* (e.g., the MW and log P o/w values of 9 phenolics active against *S. aureus* including epigallocatechin gallate, pinosylvin, and resveratrol ranged from 190 to 450 g.mol^−1^ and from 2.1 to 3.4, respectively). Elsewhere, IBHB was also selected for its low MW rendering the diffusion fast enough to design the release, for an easy detection by UV spectroscopy to study IBHB mass balance, and for its availability at an affordable cost for extrusion technology which requires rather high quantity of active substance. Meanwhile, it is to be noted IBHB is not intended to be used beyond the study (dedicated to the release properties) in the formulation of food contact materials (not in the positive list of the EU regulation (EC 10/2011)) or extended to any phenolics.

Three different films incorporating IBHB have thus been prepared with polypropylene PP, linear low density polyethylene LLDPE and ethylene vinyl acetate copolymer (EVA) from molten state to investigate the release properties from polymers usually used by industry. Conclusions of this study regarding (i) IBHB stability to the temperature conditions during extrusion and heat pressing used to prepare active films (ii) the amount and kinetics of release of IBHB from films to hydro-alcoholic solutions as food simulants should open perspectives to incorporate phenolic-rich antimicrobial plant extracts in polyolefin-based food packaging films designed to get active films progressively releasing antimicrobial phenolics to the surface of perishable foods to increase their shelf life.

## Materials and Methods

Three food grade polymeric matrices were used as received: polypropylene, PP (homopolymer, PP 505P-00900, CASRN 9003-07-0, SABIC polymers, Sittard, The Netherlands), low linear density polyethylene LLDPE [Poly (ethylene-1-butene), CASRN 25087-34-7, LLDPE 318BE, SABIC] and poly(ethylene-co-vinylacetate), EVA, 26–28% wt vinyl acetate content (EVATANE® 28.03, AK 15060122, Arkema, Colombes, France). They were all kindly provided by Addikem (Saint Pal de Mons, France). IBHB (CAS 4247-02-3, Sigma Aldrich, Saint Quentin Fallavier, France) was used as received [molecular weight 194 g.mol^−1^, log_10_ P_o/w_ 2.31, (Bouarab-Chibane et al., 2018)]. IBHB is assigned to class I according to the Cramer decision tree^2^.

### Materials Preparation

Polymeric matrices were incorporated using a IBHB/polymer ratio of 0, 1, and 2% wt) as following. Virgin polymer pellets cooled in liquid nitrogen were milled into powder using an ultra-centrifugal mill (0.5 mm sieve, Retsch ZM 200, Haan, Germany). Powders were then impregnated with a solution of IBHB in dichloromethane previously prepared in order to obtain the desired concentrations (Table 1). Dichloromethane was then evaporated during 48 h at room temperature. Pre-impregnated powders were then extruded twice using a three-zone single-screw extruder (Rheoscam, Scamex, Crosne, France), heated according to the polymer nature (see Table 1). The rotation speed of the extruder screw was set at 25 rpm. The extrusion was performed twice in order to get homogeneous stands regarding IBHB distribution, and stands were granulated with a rotating blade pelletizer (Scamex). The prepared pellets were pressed molded into films by using a constant thickness film maker mold placed in a hydraulic press (Specac, Eurolabo, Saint Chamond, France), equipped with platens heated at 140°C for LLDPE, 100°C for EVA, and 180°C for PP, a 185 bars pressure was applied for 1 min. As the duration of desorption kinetics (see section Thermal Properties) is depending on membrane thicknesses, films with different controlled thicknesses were prepared in order to get suitable durations for these experiments.

**Table 1:** Preparation of the different active pellets.

	**LLDPE**			**EVA**			**PP**		
Target IBHB/polymer ratio (wt)	0%	1%	2%	0%	1%	2%	0%	1%	2%
Polymer (g)	120	120	120	150	148.3	146.6	150	148.3	146.6
IBHB (g)	0	1.35	2.54	0	1.66	3.38	0	1.67	3.33
Percentage (%)	0	1.11	2.07	0	1.11	2.25	0	1.11	2.22
Extrusion temperatures	130/135/130°C			75/80/75°C			165/170/165°C		

*IBHB, isobutyl-4-hydroxybenzoate*.

### Scanning Electron Microscopy Observation (SEM)

Both bulk and film surfaces were observed by SEM operating at 15 kV (SEM, Hitachi TM 3030, Vélizy-Villacoublay, France). For bulk observation, films (1 day old) were broken after immersion into liquid nitrogen. For the surfaces only, films were aged during 1 week at room temperature before observation. All samples were analyzed after metallization (45 s, sputter coater with Au/Pd SC7620, Quorum technologies, Laughton, United Kingdom). All observations were made in duplicate.

### Tensile Testing

Tensile tests were performed on a texture analyzer equipped with tensile clamps (TAHD plus, Stable Micro Systems, Surrey, UK) at 50% RH and 23°C, with a crosshead speed of 60 mm.min^−1^. The films were of rectangular ribbons (typically 20^*^7^*^0.15 mm^3^). Ten to thirteen samples for each composition were tested. Young's modulus (E) were calculated form stress-strain curves considering nominal cross section of the film, as well as ultimate tensile strength (σ_B_), and strain at beak (ε_B_):

$$\begin{array}{l} \varepsilon =\frac{\text{L}-\text{Lo}}{\text{Lo}}\\ \text{E}=\frac{\sigma }{\varepsilon }and\;\sigma =\;\frac{\text{F}}{A} \end{array}$$

Where Lo and L are the initial and elongated lengths (m) of the sample, respectively, σ the tensile stress (Pa), F the tensile force (N), A the nominal cross section of the film (m^2^).

### Thermal Properties

Thermal properties of virgin polymers and of polymers formulated with isobutyl-4-hydroxybenzoate were characterized by differential scanning calorimetry (DSC). DSC analyses were performed with a DSC Q2000 apparatus (TA Instruments, Guyancourt, France). Four to six milligrams of each polymer were weighted directly in appropriate pans sealed hermetically. Samples were first heated from 0 to 150°C with a heating rate of 10°C.min^−1^, before being cooled to 0°C at 10°C.min^−1^ and heated again for a second run also undergone from 0 to 150°C at 10°C.min^−1^. The melting temperature Tm and the crystallization temperature (Tc) were taken at the onset of the peaks. All DSC runs were made in duplicate.

### Kinetics of Migration in Food Simulants

**Figure 1:**
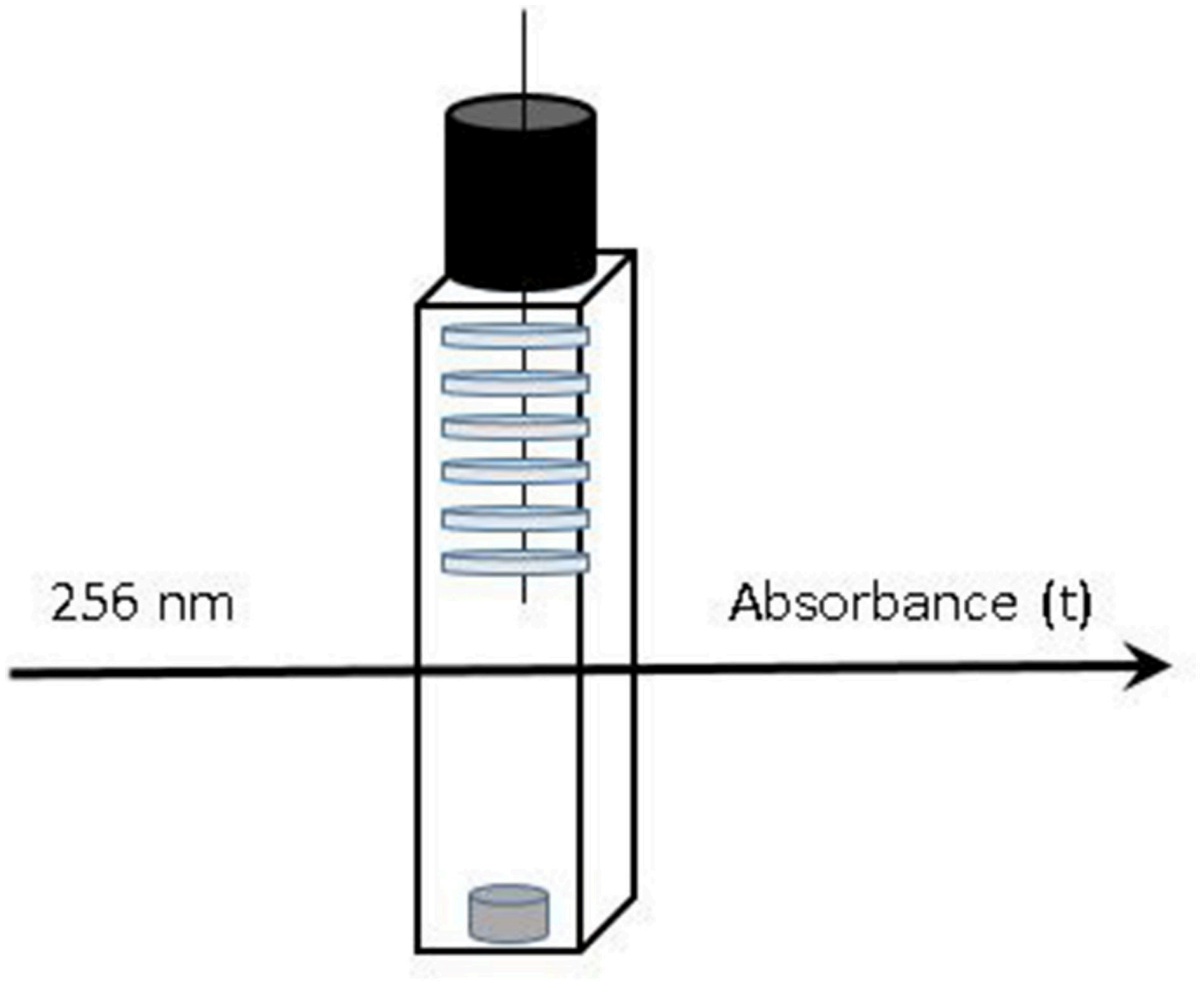
Release systems monitored vs. time: up to 6 disks of active films immersed in liquid simulant stirred in a tightly sealed quartz cell.

Films (thicknesses of 30 μm for LLDPE, 20 μm for PP and 80 or 200 μm for EVA) were placed in [95, 50, and 10% (v/v) ethanol] hydro-alcoholic solutions as food simulants in order to study the release kinetics until the equilibrium. Only one formulation (1% wt of isobutyl-4-hydroxybenzoate) was analyzed and systems were dimensioned as following (for suitable kinetics and UV saturation reasons). Films were cut with a 8 mm diameter punch to obtain 6 disks (LLDPE and PP) and only one disk was necessary for EVA films. The disks were weighed and put on a needle. The needle with films was immersed in hydro-alcoholic solutions in a quartz cell (Figure 1). The kinetics of migration were monitored as a function of time by UV spectroscopy (Biochrom libra S50, Nottingham, UK) at 256 nm until equilibrium. Systems were all weighed periodically to verify the absence of any simulant evaporation. In parallel, calibration curves [from solutions of isobutyl-4-hydroxybenzoate in 95, 50, and 10% (v/v) ethanol solutions, respectively] have been determined from Beer-Lambert law to determine precisely the amount of isobutyl-4-hydroxybenzoate released. The results were collected at least in triplicate and every release system (see Figure 1) was prepared from different part of films in order to use a representative sampling. The apparent diffusion coefficients (D) were determined from the kinetics of release data using the solution of the second Fick's law equation at short times (A_t_/A_∞_ ≤ 0.6) (Khalil et al., 2014).

$$\begin{array}{l} D=\frac{\pi \beta^{2}e^{2}}{16} \end{array}$$

where e, the sample thickness and β, the slope of the beginning of the experimental curve $\frac{A_{t}}{A_{\infty }}=f\left(\sqrt{t}\right)$ when linear *A*_*t*_ is the absorbance vs. time and *A*_∞_ the absorbance at equilibrium with respect to the Beer- Lambert law.

### Assessment of Antibacterial Activity of Films

Antibacterial activity of films was assayed following a procedure adapted from ISO 22196, 2011 standard (used to measure antimicrobial properties of a solid plastic surface) with *S. aureus* as test organism. A *S. aureus* CNRZ3 pre-culture in Mueller Hinton Broth was diluted [1/20 (v/v)] to obtain a 6.0 × 10^5^ CFU.mL^−1^ *S. aureus* suspension. Two-hundred μL of this bacterial suspension were deposited on 2.0 cm × 2.0 cm 85–90 μm thick films (with 2% wt IBHB) and subsequently covered with 1.5 cm × 1.5 cm and 30 μm width control films (without IBHB). EVA, PP, and LLDPE films were placed under UV-light for 15 min to sterilize them immediately before antimicrobial activity assays. All films tested in triplicate and cover films were incubated for 24 h at 37°C and 95% relative humidity. *S. aureus* cells present after 0 and 24 h incubation on the surface of control and IBHB-containing films were detached by placing each film in 10 mL of Dey-Engley (D/E) neutralizing broth (Grosseron, Coueron, France) and subsequent stirring for 20 s. Tween® 80 [0.3% (v/v)] was added in D/E neutralizing broth just before placing each film in D/E neutralizing broth in an ultrasonic water bath for 3 min. Films and D/E neutralizing broth were then vortexed for 1 min. The recovered *S. aureus* suspensions were serially diluted and plated on Mueller Hinton Agar. The inoculated plates were incubated for 48 h at 37°C for *S. aureus* CNRZ3 enumeration.

The antibacterial activity against *S. aureus* CNRZ3 of IBHB containing films was calculated as follows:

R = U_t_ -A_t_

U_t_: mean of culturable bacteria (log CFU.cm^−2^) detached from control films after 24 h incubation

A_t_: mean of culturable bacteria (log CFU.cm^−2^) detached from IBHB incorporated films after 24 h incubation

For EVA films only, the remaining isobutyl-4-hydroxybenzoate concentration in films was determined from migration in ethanol as described in the former paragraph (ISO 22196, 2011).

## Results and Discussion

PP, LLDPE, and EVA films incorporated with 1 and 2% wt of isobutyl-4-hydroxybenzoate or IBHB (see Table 1 for exact percentages) were elaborated by melt blending at the laboratory scale as well as control films which were made using the same procedure but without any antimicrobial additive. The elaborated films were simply named using the polymer abbreviation followed by the percentage of the introduced additive rounded to its integer part (e.g., LLDPE 0%, LLDPE 1%, and LLDPE 2% etc…). Every extrusion and film forming processed well, as expected, except for PP (with higher processing temperatures) for which slight off-odors were noticed which could be due to IBHB presence, as nothing was detected for virgin PP. It is hypothesized that this could have resulted from isobutyl-4-hydroxybenzoate evaporation or possible partial degradation at 170°C.

### Scanning Electron Microscopy Observation (SEM)

**Figure 2:**
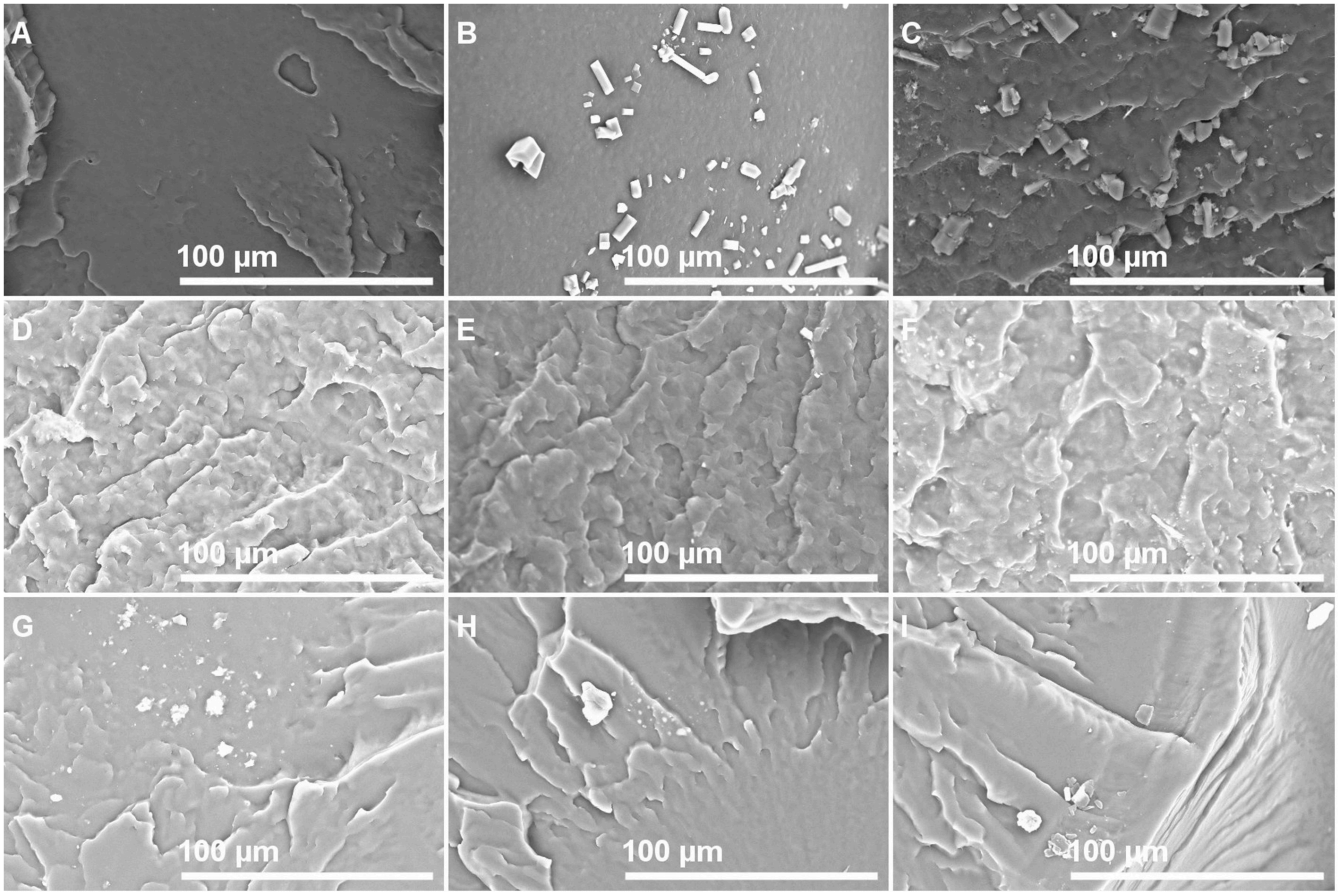
SEM observation photographs of sample cross sections: LLDPE 0% **(A)**, 1% **(B)**, 2% **(C)**; PP 0% **(D)**, 1% **(E)**, 2% **(F)**; and EVA 0% **(G)**, 1% **(H)**, 2% **(I)**.

**Figure 3:**
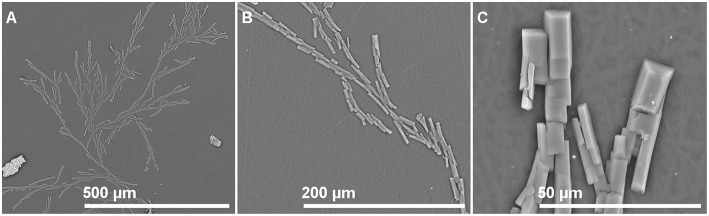
LLDPE 2%: SEM observation of the film surface after aging (1 week at room temperature). Magnification from **(A–C)**.

Photographs of films with different isobutyl-4-hydroxybenzoate contents observed by SEM in order to see if IBHB presence could be detected once embedded in the three polymeric matrices are presented in Figure 2. For this purpose, control films prepared from virgin polymers with the same procedure were also observed (Figures 2,A,D,G). For LLDPE films, small particles were observed on the materials and their presence increased with IBHB content (Figures 2B,C). For PP films (Figures 2E,F), only small spots were observed for PP 2% films. For EVA films, only surface impurities were noticed whatever the sample (EVA1% or EVA 2% films) probably because of its tackiness. These particles or spots could be attributed to IBHB, possibly crystallized considering their three-dimensional geometrical shape especially in LLDPE films. This phenomenon was related to the probable lower solubility of IBHB into PP and LLDPE than into EVA, as expected, taking into account the rather high percentages used for an additive in such semi-crystalline and apolar matrices. More precisely, the amount of IBHB probably reached the limit of solubility in LLDPE and PP 2% films. Meanwhile, these observations were achieved on cross sections of materials (after fracture following immersion in liquid nitrogen), aged for about 1 day. Following this comment, film surfaces (without previous material fracture) were also observed after 1 week of aging at room temperature for all samples. For LLDPE 2% only (Figure 3), the formation of macroscopic dendritic structures were very noticeable and explained by the spontaneous migration at the film surface of the additive over time (exudation). This phenomenon was observed at room temperature in order to simulate film storage conditions. It is due to the migration kinetics through LLDPE, which were much faster than for PP (Dole et al., 2006) to enrich quickly the material surface. In fact, this phenomenon, called blooming, is intentionally implemented for surfaces with anti-fogging or slipping agents incorporated usually at a 1–3% wt concentration in films (Plasman et al., 2005; Nouman et al., 2017). This migration at the film surface was not observed for EVA films.

### Thermal Properties

Thermal properties have been investigated considering the former results, and previously for the virgin matrices to determine the temperatures to be reached (at the laboratory scale) for compounding and thermoforming of the films at the molten state. The isobutyl-4-hydroxybenzoate melting temperature was around 76–80°C depending on its thermal history (79°C first run, 76°C for the second one). Hence, extrusion was performed from 80°C to allow the melting of the additive. The presence of IBHB tended to decrease the melting temperature of the PP matrix (from 163 to 153°C for PP, second run), but no significant modifications were reported for LLDPE and EVA (see Table 2). In conclusion, isobutyl-4-hydroxybenzoate interact with the PP matrix disturbing the crystalline organization of PP (mostly Tm and enthalpy energies).

**Table 2:** Thermal properties for virgin matrices and formulated ones with isobutyl-4-hydroxybenzoate.

	**Run 1 (first melting)**			**Crystallization**		**Run 2 (second melting)**	
	**Tm1 (°C)**	**Tm2 (°C)**	**ΔHm (J/g)**	**Tc (°C)**	**ΔHc (J/g)**	**Tm (°C)**	**ΔHm (J/g)**
Isobutyl4HydroxyBenzoate	79	–	133	–	–	76	119
LLDPE 0%	125	–	82	109	107	124	84
LLDPE 1%	124	–	87	109	113	122	84
LLDPE 2%	122	–	83	106	92	120	83
PP 0%	166	–	80	114	90	163	77
PP 1%	161	–	80	113	89	155	86
PP 2%	161	–	74	111	90	153	82
EVA 0%	46	78	a	56	51	79	27
EVA 1%	45	78	a	57	40	78	32
EVA 2%	46	77	a	57	42	79	32

*Tm, melting temperature; Tc, crystallization temperature; ΔHm for melting or ΔHc for crystallization enthalpies (if accessible)*.

*^a^Not given for a double thermal event*.

### Tensile Testing

Mechanical properties by tensile testing were performed for LLDPE and PP films (for 1 and 2% wt IBHB) which can be used as monolayer films for packaging. This study was not relevant for EVA which is always used as a sealing or adhesive layer applied onto another polymeric substrate which will ensure the basic mechanical resistance of the final packaging. Ultimate tensile strength (σ_B_), elongation at break (ε_B_), and Young's modulus (E) were given in Table 3. The results showed that the addition of the active compound (1 and 2% wt) did not modify significantly the tensile properties of PE and PP films, considering the experimental errors. These results could have been expected because of the low levels of additive incorporation of this study. Considering the experimental bars, the only change that could be reported was the Young's modulus for LLDPE 2% which increased slightly. To conclude, the mechanical performances were not affected by the incompatibility reported above which means that this incompatibility remained probably partial, especially when IBHB is introduced at the level of 1% wt. For that reason, the following release experiments have been made using that low percentage (1% wt).

**Table 3:** Ultimate tensile strength (σ_B_), elongation at break (ε_B_) and Young's modulus (E) of LLDPE and PP films when incorporated with 0, 1, or 2% wt IBHB.

**Samples**	**Strength at Break**	**Strain at break (%)**	**Young's modulus**
	**(MPa)**		**(MPa)**
PP 0%	25 ± 4	650 ± 302	861 ± 86
PP 1%	29 ± 2	856 ± 145	868 ± 80
PP 2%	30 ± 3	970 ± 86	915 ± 138
LLDPE 0%	16 ± 4	983 ± 282	160 ± 23
LLDPE 1%	14 ± 3	944 ± 204	174 ± 13
LLDPE 2%	18 ± 3	1055 ± 348	211 ± 19

### Kinetics of Release in Food Simulants

The kinetics of release of isobutyl-4-hydroxybenzoate from films were analyzed for all systems in liquid hydro-alcoholic solutions [95, 50, and 10% (v/v) ethanol], until the equilibrium. For this purpose, preliminary test systems were tested to better dimension the migration study for accurate measurements, playing both with the quantity of films for the release rates and the film thicknesses for relevant kinetics. The films used for this section were incorporated with 1% wt (exact content 1.11% wt, see Table 1). The simulants used in this study were adapted from the EU regulation (EC 10/2011) and could be assigned to aqueous foodstuffs [10% (v/v) ethanol hydro-alcoholic solution] or fatty ones [95% (v/v) ethanol hydro-alcoholic solution].

#### Isobutyl-4-Hydroxybenzoate Release Rates

The IBHB release rates (or migration rates) were gathered in Table 4. The released quantities were given in percentage (weight basis) of the theoretical amount introduced (i.e., 1.11% precisely) at equilibrium in the 3 hydro-alcoholic solutions [i.e., 95, 50, and 10% (v/v) ethanol].

**Table 4:** Isobutyl-4-hydroxybenzoate release rates in the 3 different simulants at 23°C.

**Migration rate from (%)**	**95% ethanol**	**50% ethanol**	**10% ethanol**
LLDPE	32 ± 7	33 ± 7	19 ± 3
PP	72 ± 9	82 ± 4	55 ± 5
EVA	101 ± 1	104 ± 4	72 ± 1

The release rates at equilibrium of IBHB ranged from 19 to 32% for LLDPE films from 55 to 72% for PP films, and from 72 to 100% for EVA films according to the simulants. The release rates were identical for 95 and 50% (v/v) ethanol solutions and decreased only for the simulant with the lowest ethanol content [10% (v/v)]. For EVA films, the release rates were total (100%) for these two ethanolic solutions. For PP films and especially LLDPE films, the released rates were much lower than for EVA films. Moreover, standard deviations for LLDPE and PP films immersed in 95% ethanolic solution were higher. These results are consistent with the former SEM observations which showed the heterogeneous distribution of isobutyl-4-hydroxybenzoate in LLDPE and possibly for PP films. The higher standard deviation could simply be due to the removal of IBHB (present at the film surface) during the manipulations necessary to mount the systems.

At equilibrium, isobutyl-4-hydroxybenzoate was partially available for diffusion, especially for PP and with the 10% (v/v) ethanol solution. The amount of unavailable isobutyl-4-hydroxybenzoate ranged from 28 to 45% wt of the initial quantity of incorporated additive (considering only results for PP and EVA systems). This phenomenon could be due to: (i) the presence of isobutyl-4-hydroxybenzoate aggregates shown by the SEM observations in the film bulk which could not participate readily to the diffusion. It would require for that purpose a preliminary step of dissolution induced by interactions with ethanol to render it available for diffusion. This could be probably not sufficiently achieved without substantial simulant absorption by the polymer. Indeed, the quantity of absorbed ethanol has already been measured for PP films and was reported as being around 0.5% of the initial film mass (Garde et al., 2001), which is low. This could probably limit or control the additive availability for diffusion through the own dissolution kinetic of the aggregates; (ii) the entrapment of additives in the film bulk was deeply reported by UV microscopy and was attributed to the competition between the crystallization kinetic of the polymer and the diffusion kinetic of the additive at molten state (Calvert and Billingham, 1989); (iii) a limited release due to the partition coefficient between simulant and polymer, limiting the “plateau” value reached at equilibrium, as the solubility of migrants in ethanol-water mixtures often correlates positively with increasing ethanol concentration (Seiler et al., 2014); (iv) for PP, a possible partial elimination of the additive by heat volatility or by degradation during the film elaboration, despite of the fact that UV spectra of isobutyl-4-hydroxybenzoate in the simulant remained similar to those made for calibration curves. In literature, mass balances measuring after processing the exact amounts of additives incorporated in synthetic polymers are rarely provided, because of the impossibility to solubilize polymers in convenient solvents at room temperature (for polyolefins) to assay additive concentration. Meanwhile, for EVA films, a release rate of 100% excluded any isobutyl-4-hydroxybenzoate loss. The limited release could be governed by multiple phenomena explained above, but for the simulant with 10% ethanol, the partition coefficient was certainly the most influential parameter controlling the limited release rate regarding the hydrophobic character of the additive (log_10_ P_o/w_ = 2.31).

#### Apparent Partition Coefficient

According to the precedent results, the apparent partition coefficients K_S/P_ between simulant and polymer were determined following the equation:

$$\begin{array}{l} K_{S/P}=\frac{C_{S}}{C_{P}} \end{array}$$

C_s_: additive concentration (wt/v) at equilibrium in simulant

C_p_: additive concentration (wt/v) at equilibrium in polymer

The values were given in Table 5, except for LLDPE films because of the blooming effect.

**Table 5:** Partition coefficients of isobutyl-4-hydroxybenzoate at 23°C between films and food simulants.

**Simulants and polymer matrix**	**95% ethanol**	**50% ethanol**	**10% ethanol**
PP	5 ± 4	7 ± 1	1.4 ± 0.2
EVA	∞^*^	∞^*^	2.8 ± 0.1

* *The exact value is not reachable technically and a high value of K (total release) is then identified to an infinite one*.

For EVA, isobutyl-4-hydroxybenzoate was totally available for the food (simulated with 95 and 50% (v/v) ethanol) and the apparent partition coefficient K_S/P_ dropped to about 3 for 10% ethanolic solution. For PP, the apparent partition coefficient (5 ± 4) was scattered for 95% (v/v) ethanol solutions but seemed to decrease to about 1 for 10% (v/v) ethanol solutions. The apparent partition coefficients in Table 5 could be compared with a study dedicated to the correlation of foodstuffs with ethanol-water mixtures with regard to the solubility of migrants, based on LDPE as model of polyolefins (Seiler et al., 2014). These authors proposed a quasi linear correlation between Log 1/K and Log P_o/w_, useful to calculate K values for any food of interest via the assigned ethanol-water equivalency. Indeed, the prediction of K by calculation gives the release rate without waiting for the equilibrium. They identified a threshold of log P_o/w_ of 2 for the additives. Below this level, additives cannot be incorporated in polyolefins because of compatibility reasons. From their analysis, with a log P_o/w_ of 2.31, close to this threshold, isobutyl-4-hydroxybenzoate would be partially compatible with polyolefins (LLDPE and PP) and the release rates at equilibrium should be equivalent for the simulants containing from 20 to 95% ethanol and very limited below 20%, considering the log scale they proposed (Seiler et al., 2014). Our results are in a good agreement with these assumptions.

#### Kinetics of Isobutyl-4-Hydroxybenzoate Release

**Figure 4:**
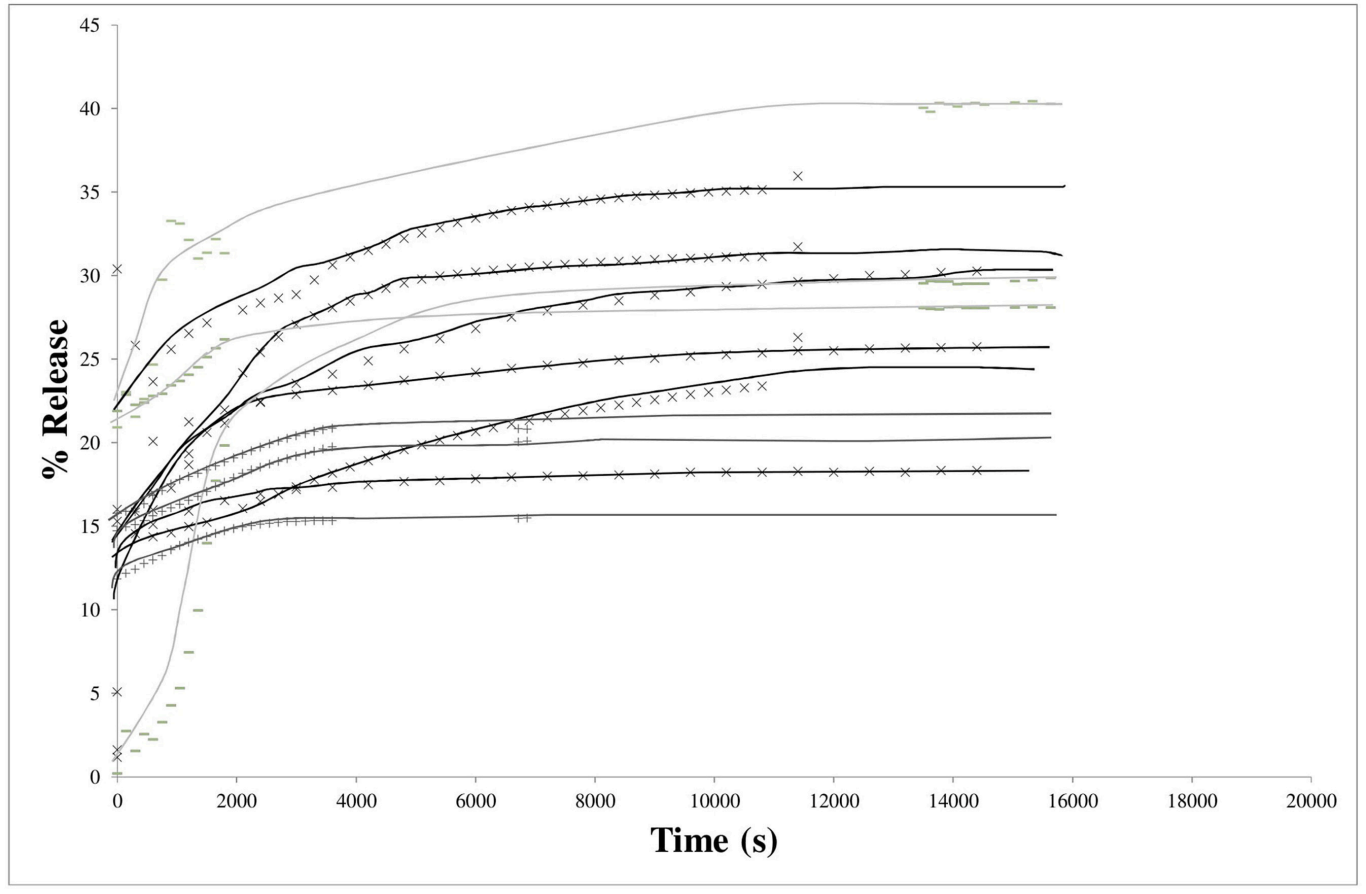
Kinetics of isobutyl-4-hydroxybenzoate release from LLDPE films (thickness 30 μm) in the 3 different hydro-alcoholic simulants (× 95% ethanol; −50% ethanol; + 10% ethanol).

In Figure 4, raw data for the LLDPE films (9 similar systems) were given. Obviously, the release proceeded in two steps: Y-intercepts were not equal to zero, as attempted, but were varying readily from 2 to nearly 25%, followed by curves demonstrating the diffusion from the bulk as a function of time. Y-intercepts and plateau values (at equilibrium) were scattered. Once again, these results confirmed the presence at the surface of IBHB dissolved when immersed to contribute to the global release rate previously to the diffusion phenomena from the bulk. The equilibrium was reached nearly after 4 h for 30 μm thick films.

**Figure 5:**
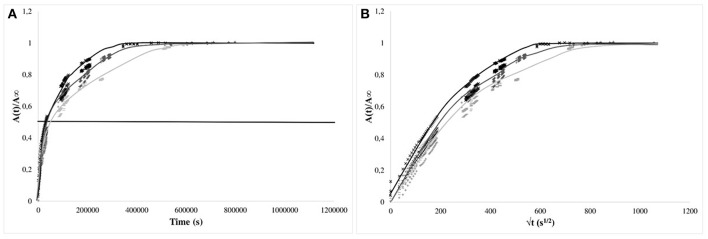
Kinetics of isobutyl-4-hydroxybenzoate release from PP films (20 μm) in the simulants (× 95% ethanol; −50% ethanol; + 10% ethanol) vs. time **(A)** and square root of time **(B)**. The horizontal bar is given as guide line to compare the half times of release.

**Figure 6:**
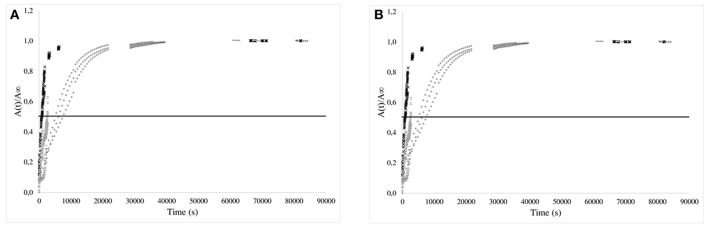
Kinetics of isobutyl-4-hydroxybenzoate release from EVA films (80 or 200 μm) in the 3 simulants (× 95% (v/v) ethanol; −50% (v/v) ethanol; + 10% (v/v) ethanol) vs. time **(A)** and normalized square root of time **(B)**. The horizontal bar is given as guideline to compare the half-times of release.

For PP and EVA films, the values were normalized, as usually proposed, with respect to the equilibrium values and presented as a function of time (Figures 5, 6, right) and square root of time (Figures 5, 6, left). For PP films, the kinetics of release were much slower than for 30 μm thick LLDPE films but approximately similar whatever the nature of the simulant: the time required to reach one-half of the total normalized release was about 7–8 h for 20 μm thick films. This time is to be compared to the time necessary to reach the equilibrium (between 100 and 170 h). For these systems, diffusion phenomenon through the polymer controlled the kinetics of transfer. However, the behaviors for some systems deviated, within the experimental uncertainties, from a Fickian behavior, possibly because of migration from embedded aggregates acting as reservoirs partially available and/or because of the problem of systems aging during immersion as the release lasted for about 7 days.

For EVA films, the isobutyl-4-hydroxybenzoate release kinetics were clearly dependent on the nature of the simulating media and were more rapid. The time to reach 50% of transfer equilibrium were approximately only 30 to 40 min for the thickest films (200 μm) (respectively immersed in 95 and 50% (v/v) ethanol solutions (Figure 6, left) and was about 100 min for the thinnest films (80 μm) once dipped in 10% (v/v) ethanol solutions. When normalized with the film thicknesses, it is obvious that the transport is accelerated when the percentage of ethanol increased (Figure 6, right). This phenomenon was related to the plasticization of EVA by ethanol which activated therefore the transport of the additive (Vicente and Gottifredi, 2001).

The apparent diffusion coefficients of isobutyl-4-hydroxybenzoate were determined at short times of diffusion only when immersed in 95% (v/v) ethanol, keeping only the kinetics having a Fickian behavior [i.e., 95% (v/v) ethanol solutions]. Apparent diffusion coefficients of IBHB through EVA1% and PP1% were 2.8 ± 0.3 × 10^−12^ m^2^s^−1^ and 4 ± 1 × 10^−16^ m^2^s^−1^, respectively. It should be noted that four orders of magnitude separated EVA from PP. If the values obtained for the EVA seemed comparable to the values of the literature (Dole et al., 2006), the apparent diffusion coefficient in polypropylene seemed to be low. This could be relied to the possible superimposed slow dissolution of isobutyl-4-hydroxybenzoate aggregates in the bulk.

#### Antibacterial Activity Assay of Isobutyl-4-Hydroxybenzoate-Incorporated Polyolefins Films

**Figure 7:**
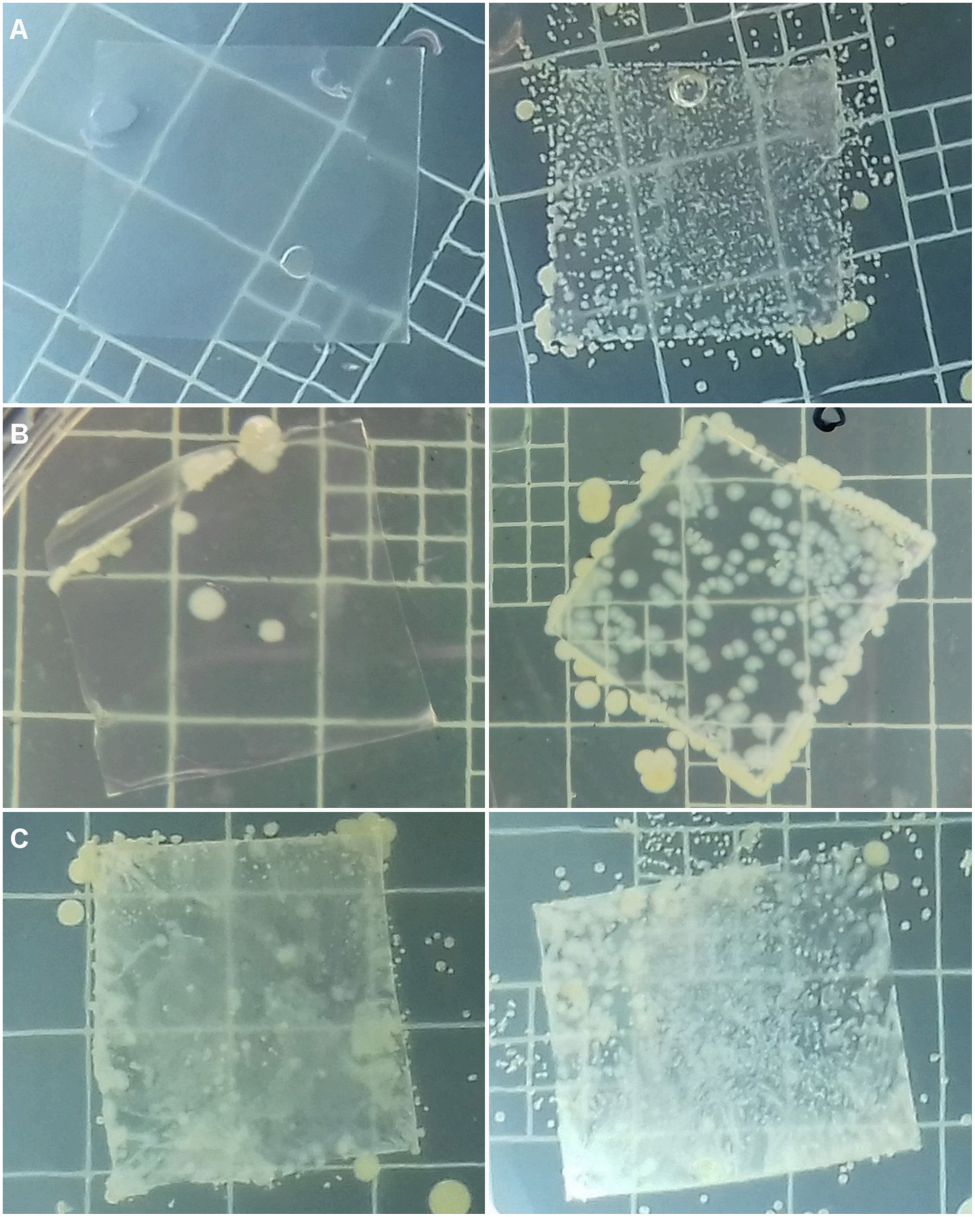
Photographs of LLDPE2% **(A)**, EVA2% **(B)**, et PP2% **(C)** films and corresponding control films without isobutyl-4-hydroxybenzoate in the right column, inoculated with *S. aureus* (6.0 × 10^5^ CFU mL^−1^) after 48 h incubation at 37°C on MHA agar, previously recovered after a 24 h incubation at 37°C and 95% relative humidity.

The antibacterial activity of films was assayed, in order to assess if films were still active after all the different processing stages necessary to make films. The methodology was adapted from standard methods and relied upon a simple contact during 24 h at 37°C to check whether the *S. aureus* strain tested is able to grow on film surface (Figure 7). An enumeration was performed (Table 6). The results collected for films prepared with the 3 polymer matrices (EVA, LLDPE, and PP) incorporated with 2% (wt/wt) IBHB are presented in Table 6. This high IHB concentration was chosen to enhance the test sensitivity.

**Table 6:** Anti-*Staphylococcus aureus* CNRZ3 activity for EVA, LLDPE, and PP films incorporating isobutyl-4-hydroxybenzoate (2%, wt/wt) (*n* = 3).

**Polymer matrix**	**R: Log_**10**_ reduction of *S. aureus* (CFU.cm^**−2**^)**
LLDPE	6
EVA	4
PP	1

*The film surfaces were 4 cm^2^ and thicknesses were equal to 85 μm for LLDPE and PP, 90 μm for EVA films*.

The value of antibacterial activity of films with 2% wt IBHB of 6 indicates that after 24 h, a 6 log_10_ CFU.cm^−2^ reduction of *S. aureus* cells culturable population detached from these films compared to the *S. aureus* population detached from control films. This demonstrates the possibility to prepare active LLDPE-films containing antimicrobial phenolics by extrusion. These results ranked LLDPE films incorporating 2% (wt/wt) IBHB at the top of the anti-*S. aureus* activity with a 6 log_10_ CFU.cm^−2^ reduction of *S. aureus* culturable cells, followed by a 4 log_10_ CFU.cm^−2^ reduction for EVA films and about a 1 log_10_ CFU.cm^−2^ reduction for PP films. In parallel, in Figure 7, no growth of *S. aureus* CNRZ3 on LLDPE films was observed in Figures 7A,B, compared to the corresponding control films.

In Table 6, PP films containing 2% (w/w) IBHB exhibited the weakest antibacterial activity against *S. aureus* compared to the films made with the other two polymer matrices. Several reasons can explain this difference: (i) the highest temperatures in extrusion (170°C) and hot press (180°C) for the preparation of films, (ii) a possible loss of IBHB by evaporation during the extrusion step, (iii) and mainly the slowest release properties which could only correspond to the low amount of IBHB present on the surface of the film as the additive present in the bulk was probably not released in due time to act effectively against *S. aureus*. For EVA films, the release kinetics of IBHB were much faster compared to PP films (i.e., Four orders of magnitude higher when immersed in 95% (v/v) ethanol) and the diffusion from the film bulk could have effectively contributed to the film antibacterial activity. Quantitatively, a target concentration at least equivalent to the minimal bactericidal concentration (MBC) (previously measured ≤ 1 g L^−1^) (Bouarab-Chibane et al., 2018) of IBHB against *S. aureus* can be targeted. During the antimicrobial activity assays, such a concentration has been potentially reached for LLDPE films because of the presence at the surface of IBHB immediately available. For PP films, the release could be very low because of kinetic reasons.

For EVA films, the determination of the remnant concentration in the film was possible. Indeed, after the contact with the broth (37°C, 48 h), an extraction of the films with 95% (v/v) ethanol was performed until the equilibrium, as previously described. The results gave 50% ± 5% of remnant concentration, which corresponded to a former released concentration of IBHB of 2 g.L^−1^ (extracted by the broth), that is above *S. aureus* CNRZ3 MBC.

## Conclusion

In this study, the design and release of antimicrobial systems, based on polyolefinic films (LLDPE, PP, and EVA) incorporating 1 or 2% wt of isobutyl-4-hydroxybenzotate (IBHB) were investigated. IBHB was selected as a model phenolic migrant to especially study the release properties. The release was studied in ethanol-water mixtures [95, 50, and 10% (v/v) ethanol] simulating foodstuffs (adapted from the EC 10/2011 regulation).

Interestingly, antimicrobial activity was preserved despite of both thermal and mechanical treatments applied during film elaboration, especially for LLDPE and EVA films.This demonstrates the possibility to prepare active films based on LLDPE or EVA containing antimicrobial phenolics such as IBHB by extrusion at high temperature. In future studies, it will thus be possible to integrate instead of IBHB other active phenolics that can be chosen in connection with their antimicrobial activity spectrum. It is even possible to imagine as original applications, food products like probiotic yogurt/curd having live and active microbial culture, antimicrobial plant phenolics or plant extracts that selectively inhibit pathogenic bacteria, while allowing the growth of probiotic bacteria at the same concentration can be selected, as recently suggested (Pacheco-Ordaz et al., 2017).

Different behaviors were observed depending on the matrices. Isobutyl-4-hydroxybenzotate was not fully compatible with LLDPE (1 and 2% wt) and PP (especially 2% wt) and the so-called blooming phenomenon was only observed for LLDPE when incorporated with 2% wt. EVA films were homogeneous whatever the IBHB percentages, at the scale of the observation, which was expected for a semi-polar copolymer with a high level of vinyl acetate. The release behaviors were thus studied only with films containing 1% wt of IBHB to limit the compatibility issue.

For PP, the release of isobutyl-4-hydroxybenzoate was too slow for the purpose of preserving fresh food and off-odors were of concern. For LLDPE films, the kinetics of diffusion were suitable but perturbed by the blooming effect (immediate dissolution of isobutyl-4-hydroxybenzotate at the surface from which quantity depended on the age of the films) followed by delayed diffusion from the bulk. For EVA films, the speed of release was too fast. For all systems, the release rates were lower when the ethanol concentration of the simulant was the lowest [10% (v/v)].This was attributed mainly to the partition coefficient, the additive being less soluble in water than in ethanol. The kinetics of release of isobutyl-4-hydroxybenzoate were similar whatever the simulant for PP films, unlike for EVA films. For this last material, a plasticization effect due to concomitant ethanol sorption greatly accelerated the release.

**Figure 8:**
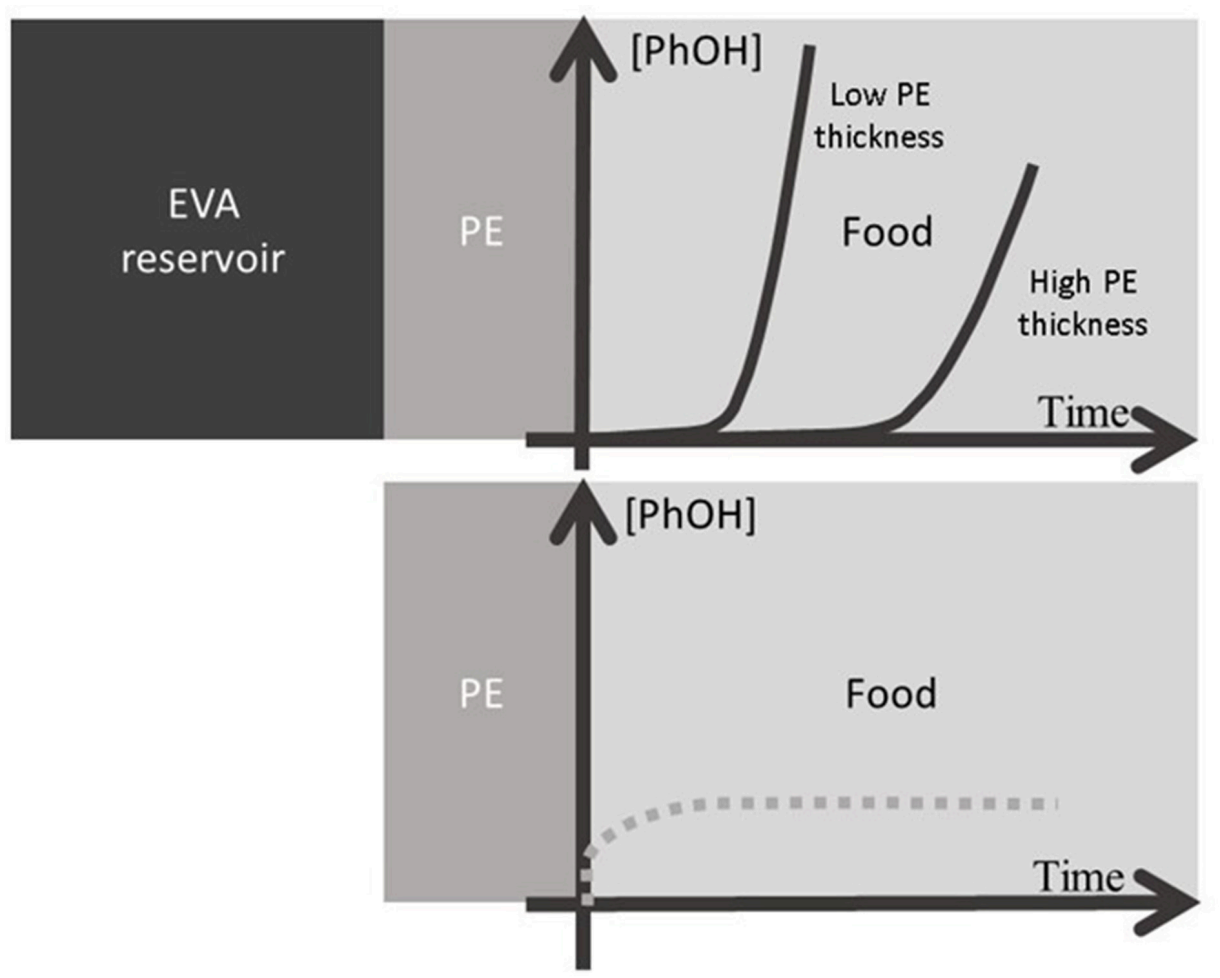
Schema of multilayer polyolefin based packaging for the controlled release of active additives: EVA is the reservoir layer of different capacities playing with the copolymerization rate, and the additive permeation through the PE layer controlled the flux (full line) playing with its thickness. The dotted line represents the migration from direct contact with a layer incorporated with additives.

As a conclusion, none of these three polymers is actually suitable for a suitable release kinetic compatible with perishable food but a combination of two of them PE and EVA are promising. In fact, active packaging films incorporated with antimicrobial additives suffer from a lack of active substance always limited by the low volume of packaging playing the role of a limited “reservoir”—compared to the quantity of food to be packed. As a perspective, in order to increase the effectiveness over the time of antimicrobial packaging films, the use of co-extruded multilayer films could be tailor-made. The inner layer of the film could be LLDPE, possibly with no antimicrobial additive, acting as a “barrier” layer and the other layer(s) could be made of EVA copolymers acting as reservoirs of different capacities and release kinetics playing with the level of vinyl acetate. According to the thicknesses of each layer and level of incorporation, both quasi immediate diffusion and a delayed one can be achieved in order to regulate the flux of additives over a required period of time. The Figure 8 illustrates this release.

The perspectives of this work will be now focused on phenolic compounds extracted from plants in order to design active packaging films based on LLDPE and EVA.

## Data Availability

All datasets generated for this study are included in the manuscript and/or the supplementary files.

## Author Contributions

AC managed daily the experimental part related to the transport phenomenon, prepared the figures and run calculations. LB managed the experimental work related to the antimicrobial activity of films as Ph.D. student. JD was supervised by AC during her internship period in the laboratory. NO contributed to select the contents, supervised the section about antimicrobial activity of films, SEM observations as well as the preliminary state of the art, based on her expertise based on food microbiology. PD headed the ANR project referenced below, contributed to select the contents and provide expertise about active antimicrobial system selection, food and analytical sciences. CJ conceived and wrote the manuscript from her expertise about polymer sciences, transport phenomena and food packaging.

### Conflict of Interest Statement

The authors declare that the research was conducted in the absence of any commercial or financial relationships that could be construed as a potential conflict of interest.

## Footnotes

^1^Commission Regulation (EU) No 358/2014 of 9 April 2014 amending Annexes II and V to Regulation (EC) No 1223/2009 of the European Parliament and of the Council on cosmetic products (OJ L 107, 10.4.2014), *p*. 5.

^2^(https://apps.ideaconsult.net/data/ui/toxtree) Accessed February 13, 2019.
